# Identification of genetic factors that modify motor performance and body weight using Collaborative Cross mice

**DOI:** 10.1038/srep16247

**Published:** 2015-11-09

**Authors:** Jian-Hua Mao, Sasha A. Langley, Yurong Huang, Michael Hang, Kristofer E. Bouchard, Susan E. Celniker, James B. Brown, Janet K. Jansson, Gary H. Karpen, Antoine M. Snijders

**Affiliations:** 1 Life Sciences Division, Lawrence Berkeley National Laboratory, Berkeley, CA, USA; 2 Biological Sciences Division, Pacific Northwest National Laboratory Richland, WA, USA; 3 Department of Molecular and Cell Biology, University of California, Berkeley, CA, USA

## Abstract

Evidence has emerged that suggests a link between motor deficits, obesity and many neurological disorders. However, the contributing genetic risk factors are poorly understood. Here we used the Collaborative Cross (CC), a large panel of newly inbred mice that captures 90% of the known variation among laboratory mice, to identify the genetic loci controlling rotarod performance and its relationship with body weight in a cohort of 365 mice across 16 CC strains. Body weight and rotarod performance varied widely across CC strains and were significantly negatively correlated. Genetic linkage analysis identified 14 loci that were associated with body weight. However, 45 loci affected rotarod performance, seven of which were also associated with body weight, suggesting a strong link at the genetic level. Lastly, we show that genes identified in this study overlap significantly with those related to neurological disorders and obesity found in human GWA studies. In conclusion, our results provide a genetic framework for studies of the connection between body weight, the central nervous system and behavior.

Impaired motor function is associated with a broad spectrum of human conditions including neurodegenerative diseases, and neurological and neurodevelopmental disorders. For example, Parkinson’s disease (PD), the second most common neurodegenerative disease, is characterized by severe and progressive motor impairment^1^,^2^. In addition, accumulating evidence indicates that motor impairment is a common feature of early Alzheimer’s disease (AD)^3^,^4^. Motor deficits ranging from fine to gross motor skills have been observed in subsets of children diagnosed with autism spectrum disorder or attention-deficit hyper-activity disorder (ADHD)^5^. However, motor function is multifactorial and depends on the coordinated activity of motor control systems in the brain, the spinal cord, the peripheral nervous system and the musculoskeletal system. Not surprisingly, the genetic risk factors that may contribute to many of these conditions remain poorly understood.

The discovery of susceptibility genes is facilitated in genetic reference populations with assembled genomes that allow dissection of the complex interactions that underlie the roles of genes in disease. The identification of high penetrance genes that confer genetic predisposition in certain rare human families has been successful, but it is likely that low penetrance genes present at high frequency in the human population are major genetic components associated with disease risk and many complex phenotypic traits. Recent biotechnological advances paved the way for large-scale genome-wide association (GWA) studies to identify genetic loci that contribute to human phenotypes. GWA studies have identified many loci and genes associated with numerous diseases and phenotypic traits, including body weight, obesity and neurological disorders^6^,^7^,^8^,^9^. However, disease risk often depends on life history, specifically interactions between genetic background and the environment^10^,^11^,^12^,^13^. Diet, lifestyle, chemical exposures, and other confounding factors are difficult to control in human populations. Thus, GWA studies often require enormous sample sizes to identify significant genetic associations, and still provide no guarantees that the most important loci will be discovered.

Model systems offer many advantages for the study of the genetic basis of complex traits because both genetic and environmental components of risk can be specified and tightly controlled. Studies in mouse strains with diverse genetic backgrounds and known genetic structures offer unprecedented opportunities to identify genetic loci that contribute to traits of interest, and to investigate mechanistic contributions. “The Collaborative Cross (CC)”, a large multi-parental panel of recombinant inbred strains, recently became available^14^,^15^,^16^,^17^. The CC consists of a population of mice with genetic and phenotypic diversity on par with the human population^15^. This resource, which was established by combining the genomes of eight diverse founder strains (A/J, C57BL/6J, 129S1/SvImJ, NOD/LtJ, NZO/HlLtJ, CAST/EiJ, PWK/PhJ, and WSB/EiJ), captures nearly 90% of the known variation present in laboratory mice.

We leveraged the CC and the commonly used rotarod test to generate measurements of a complex phenotype with neurological components. Locomotor function and motor coordination depend on balance, muscle strength and non-motor factors including body weight skeletal traits (*e.g.* body, tail and foot length), aggressiveness, motivation and fear of falling. Differences have been reported in motor ability among different inbred strains of mice, and correlation analyses between weight and rotarod performance was observed for certain strains^18^,^19^; however, other studies reported a lack of correlation between body weight and rotarod performance^20^. Previously, a F2 mouse intercross study (129/S6 × C57BL/6) identified quantitative trait loci (QTL) associated with rotarod performance^21^. This study identified two separate QTL affecting performance, but did not analyze the effect of body weight on rotarod performance. One of the limitations of F2 mapping studies is that only genomic loci that vary between the two target strains are interrogated by linkage analysis. The strength of using the CC mice is that genetic variation is distributed densely across the genome.

Here we identified genetic loci associated with motor performance and body weight using the CC mice. We observed that rotarod performance and body weight are highly variable among the CC strains. Linkage analysis revealed many loci contributing to rotarod performance and body weight and, importantly, a significant overlap of genetic linkage demonstrating an inverse correlation between these two phenotypes. Finally, we identify numerous candidate genes in CC quantitative trait loci with orthologs that are associated with obesity and neurodegenerative disorders in human populations.

## Results

### Rotarod performance covers a wide range in Collaborative Cross mice

**Figure 1:**
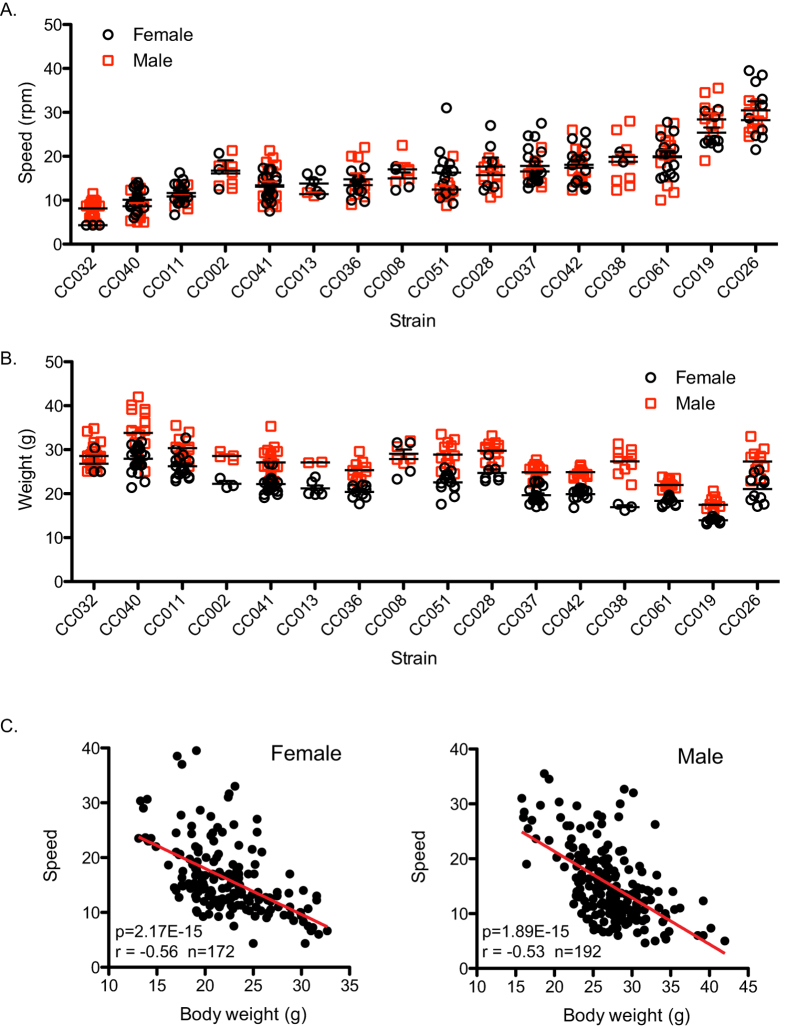
Rotarod performance and body weight are variable in Collaborative Cross mice. (**A**) Rotarod performance was measured using the accelerating rotarod test (20 rpm/min) in 16 CC strains at 10 weeks of age. The speed at animal fall is indicated for male (blue open squares) and female mice (black open circles). (**B**) Body weight of 16 CC strains at 10 weeks of age is shown for male (blue open squares) and female mice (black open circles). (**C**) Rotarod speed at the time of falling is significantly correlated with body weight in both female (left; r = −0.56, p = 2.17E-15) and male (right; r = −0.53, p = 1.89E-15) mice.

Rotarod performance was measured in a total of 365 mice (173 female; 192 male) from 16 CC strains at 10 weeks of age, with at least eight mice tested for each strain (N = 8 to 42 mice; Table S1). Mice were individually placed on a slowly rotating rod (4 rpm/minute), subjected to continuous acceleration set at 20 rpm/minute, and the speed at which the mouse fell off the rod was recorded. The average rotarod performance (average speed at the time of fall) for male and female mice of each strain was highly correlated (Pearson r = 0.94, p < 0.0001; Figure S1). However, rotarod performance varied widely among different CC strains, strongly suggesting the influence of genotype (Fig. 1A; Table S1). The average speed at the time of fall ranged from 4.3 rpm (CC032) to 30.5 rpm (CC026) in female mice, and 8.1 rpm (CC032) to 28.4 rpm (CC019) in male mice.

### Body weight is significantly correlated with rotarod performance

Some studies have reported a significant correlation between body weight and rotarod performance^18^,^19^. To investigate whether this relationship is maintained in a genetically diverse mouse population, we measured body weight of all mice on the day of rotarod testing (10 weeks of age). Similar to rotarod performance, the average body weight for male and female mice of each strain was highly correlated (Pearson r = 0.86, p < 0.0001; Figure S1), but displayed a wide range among different CC strains (Fig. 1B; Table S1). Average body weight in female mice ranged from 13.1 g for CC019 to 27.9 g for CC040, and from 17.5 g for CC019 to 33.8 g for CC040 in male mice. Importantly, we observed a significant inverse correlation between body weight and rotarod performance in male (Fig. 1C; p = 1.89E-15, Spearman r = −0.53) and female mice (Fig. 1C; p = 2.17E-15, Spearman r = −0.56). We conclude that higher body weight is significantly correlated with decreased rotarod performance, and that both phenotypes are affected by genetic background.

### Genetic linkage analysis of rotarod performance and body weight

**Figure 2:**
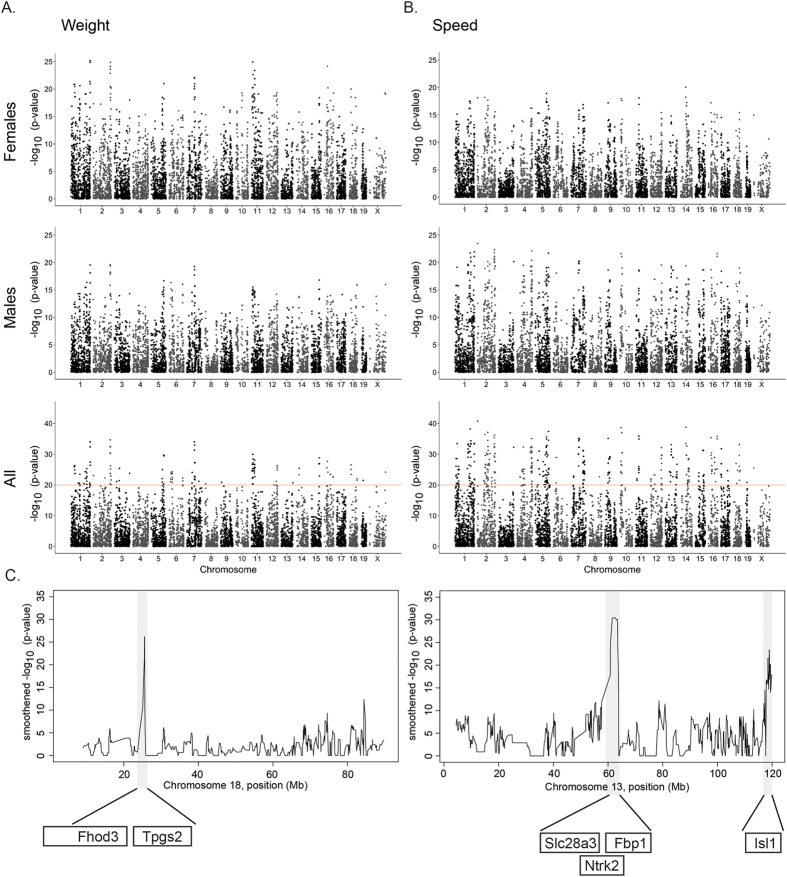
Linkage analysis identified genetic loci associated with rotarod performance and body weight. (**A**) Genome wide linkage is shown for body weight in female (top) and male (middle) mice separately and combined (bottom). The −log(p-value) is shown for 20,199 SNPs ordered based on genomic position. (**B**) Genome wide linkage is shown for rotarod performance in female (top) and male (middle) mice separately and combined (bottom; the horizontal red line indicates the QTL significance threshold at –log(p-value) = 20). The –log(p-value) is shown for 20,199 SNPs ordered based on genomic position. (**C**) Chromosome specific linkage is shown for chromosomes 18 (body weight, left) and 13 (rotarod performance, right) and only genes are listed that show association with human body weight and neurological disorders by GWAS (see Fig. 4).

To identify the genetic loci associated with rotarod performance or body weight we performed independent quantitative trait locus (QTL) analyses using 20,199 informative SNPs across the genome. Significant linkage was defined as –log(p-value) > 20 (−log(FDR) > 18), and suggestive linkage was defined as 10 ≤ –log(p-value) ≤ 20 (8 ≤ −log(FDR) ≤ 18). This analysis revealed a complex association of QTL across the genome with rotarod performance or body weight (Fig. 2; Table S2). Linkage profiles were highly correlated between male and female mice (Fig. 2A,B), so we combined male and female data for each strain to determine significant loci associated with body weight or rotarod performance.

**Figure 3:**
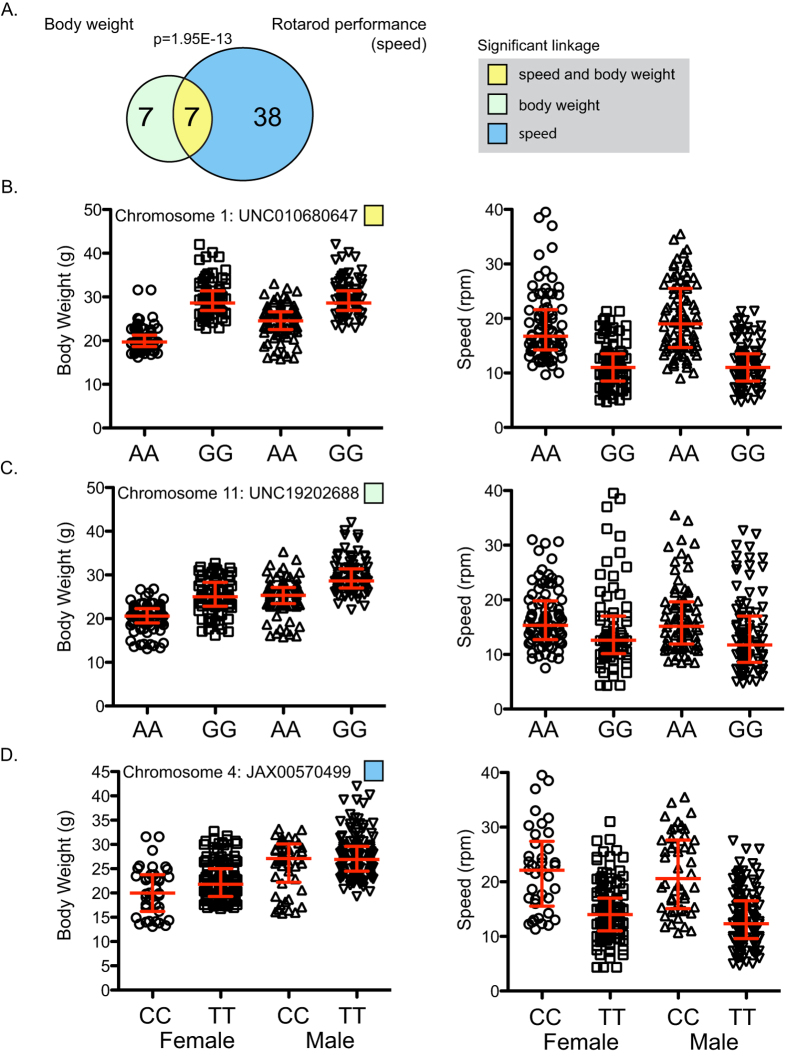
Relationship between genetic loci affecting rotarod performance and body weight. (**A**) Overlap of fifty-two loci that showed significant linkage with body weight and/or rotarod performance (speed) (p-value for overlap = 1.95E-13). (**B**) SNP UNC010680647 on chromosome 1 is significantly associated with body weight (left) and rotarod performance (right) in male and female mice. (**C**) SNP UNC19202688 on chromosome 11 is significantly associated with body weight (left), but not rotarod performance (right) in male and female mice. (**D**) SNP JAX00570499 on chromosome 4 is significantly associated with rotarod performance (right), but not body weight (left) in male and female mice. Error bars represent median and interquartile range.

**Figure 4:**
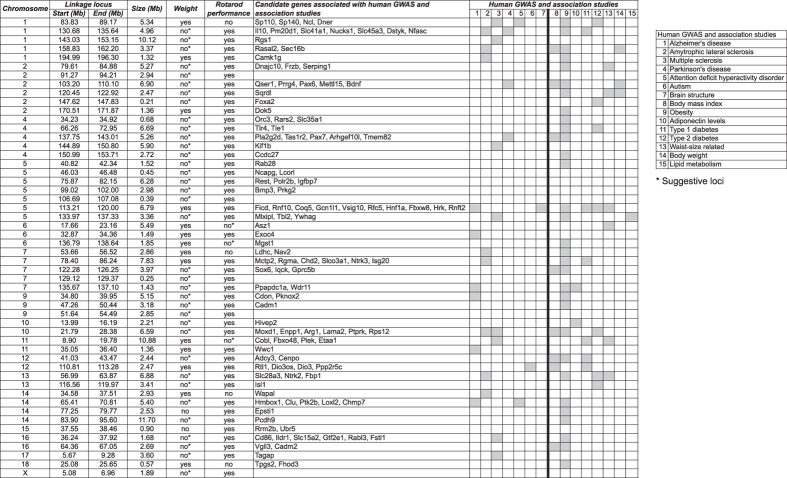
Genetic loci associated with body weight and rotarod performance. Genomic locations of QTL affecting body weight and rotarod performance. Candidate genes within QTL associated with human genome-wide association studies of neurological and body weight related disorders and traits are listed. For each locus, human GWAS and association studies are indicated in gray when genes identified in these studies show overlap with rotarod performance and body weight candidate genes.

Importantly, genetic loci for rotarod performance significantly overlap with those that control body weight, and display an inverse correlation (Fig. 3A). We identified 45 significant loci associated with rotarod performance, and 14 significant loci associated with body weight. Importantly, 7 of these loci showed significant association with both rotarod performance and body weight (p-value of overlap 1.95E-13; Figs 3A and 4 and Tables S2 and S3). All 7 loci associated with body weight and rotarod performance were inversely correlated; none showed association with increased body weight and increased rotarod performance. Examples of the classes of linkage data are shown for three individual SNPs in Fig. 3A–C. The major pattern observed is reflected in the GG allele at SNP UNC010680647 on chromosome 1, which is significantly associated with increased body weight (−log(p) = 27.5) and decreased rotarod performance (−log(p) = 33) (Fig. 3B). On the other hand, the GG allele at SNP UNC19202688 on chromosome 11 is significantly associated with increased body weight (−log(p) = 22.2), but not rotarod performance (−log(p) = 3.5) (Fig. 3C). Conversely, the TT allele at SNP JAX00570499 on chromosome 4 is significantly associated with rotarod performance (−log(p) = 25.6), but not body weight (−log(p) = 2.2) (Fig. 3D).

We then asked if QTL significantly associated with body weight exceeded the suggestive linkage threshold (10 < −log(p-value) < 20) for rotarod performance, and vice versa. We found that 3 out of 7 body weight QTL showed suggestive linkage for rotarod performance, and 36 out of 45 rotarod performance QTL showed suggestive linkage for body weight (Table S2). The extensive overlap between body weight and rotarod performance QTL for both significant and suggestive loci indicates a strong link at the genetic level. It should be noted that additional suggestive loci associated with either body weight or rotarod performance alone are likely to exist. In addition, we identified 4 loci that were associated exclusively with body weight and not rotarod performance, suggesting that other genotypic differences can override the strong inverse correlation between body weight and neuromuscular capabilities (Fig. 4).

### Rotarod performance and body weight candidate genes show significant overlap with human GWAS for neurological disorders and obesity

To discover candidates genes that could impact body weight and rotarod performance, we first compiled a list of all genes contained within the boundaries of the 52 significant QTL identified as affecting these phenotypes in the linkage analysis (n = 1694 genes; Table S3). We then compared these candidate mouse genes to a compiled list of genes (n = 1776 genes) previously associated with human body weight, diabetes and neurological disorders in GWA studies (n = 268, GWAS listed in Table S4)^22^,^23^. We found that 107 mouse genes contained within 38 of 52 QTL showed overlap with at least one human disease-associated gene (p = 1.33E-12 for the overlap between mouse genes and human GWAS identified genes) (Fig. 4, S2). An additional six candidate genes (FOXA1, DOK5, ISL1, WWC1, RRM2B and UBR5) were found in five QTL that were identified in human association studies of type-2 diabetes, Alzheimer’s and Huntington’s disease (Fig. 4)^24^,^25^,^26^,^27^,^28^. We observed that many genes contained within QTL were associated with both body weight and neurological disorder related human genes (n = 18 of 43 QTL), and 6 and 19 QTL contained genes associated with only neurological disorders or body weight related genes, respectively.

## Discussion

In this study, we identified genetic loci associated with motor performance and body weight using CC mice. Our results show that rotarod performance and body weight are highly variable among different strains of the CC, demonstrating that the Collaborative Cross provides an excellent platform for studying interactions between body weight, neurological disease and genotype. Using linkage analysis we identified 45 and 14 genetic loci contributing to rotarod performance and body weight, respectively. We observed a significant overlap between loci affecting rotarod performance and body weight (7/45 rotarod QTL and 7/14 body weight QTL), and in all cases of overlap high body weight was associated with low rotarod performance. At this time we cannot determine if these genetic loci coordinately control both body weight and rotarod performance, if the primary effect is reduced neuromuscular activity which results in increased body weight, or if they directly affect body weight which leads to reduced neuromuscular activity and rotarod performance.

The existence of some alleles associated with only rotarod performance or body weight, but not both, indicates that the rotarod test quantifies aspects of the motor phenotype beyond body weight. We observed two genetic loci exclusively associated with rotarod performance, on chromosomes 14 and 15 (Fig. 4). On chromosome 15, the identified QTL spans a region <1 Mb in size and contains seven genes, including Rrm2b and Ubr5. Orthologs of these genes were identified in a recent GWA study of Huntington’s disease clinical onset^28^, indicating that rotarod tests capture neurological traits important to human health. Rrm2b knock-out mice exhibit attenuated dNTP pools and severe mitochondrial DNA (mtDNA) depletions, functions previously associated with Huntington’s disease^29^,^30^. Additionally, a study aimed at mapping strain differences in locomotor activity identified two QTL that control rotarod performance^21^. There, F1 hybrids (129S6/SvEvTac × C57BL/6J) were intercrossed to generate the F2 generation, which was assessed for motor performance on four consecutive days. No QTL were found for rotarod performance on day 1, one QTL associated with longer latency to falling was found on day 2 (D2MIT224; 129S6/SvEvTac allele) and one QTL associated with longer latency to falling on day 3 (D1MIT270; C57BL/6J allele). We identify a genome-wide significant SNP (−log(p) = 15.3) on chromosome 1 (SNP UNC2191344) for rotarod performance, but not body weight, corresponding to the QTL at D1MIT270, thus confirming this result. There is substantial genetic variation among substrains of 129, which could explain the fact that we did not detect the QTL at D2MIT224. This seems likely since the 129 substrain used in the F1 intercross study is distinct from the one used to generate the CC. Also, our study did not incorporate a learning component across multiple days, and instead consisted of 3–5 consecutive trials all on the same day. Thus, it is possible that the QTL identified on day 2 and 3 of rotarod testing in^21^ are also associated with learning.

We identified 52 genetic loci associated with either body weight and/or rotarod performance. We were able to identify candidate genes in QTL by comparing to human GWAS and other association studies for 43 out of 52 QTL. For 9 loci, we were not able to identify candidate genes associated with human body weight or neurological disorders. There are several possible explanations. The most obvious is that rotarod performance is a complex phenotype depending on many factors in addition to body weight and motor impairment associated with neurological disorders, and it is possible that we identified genetic loci that control body weight and rotarod performance in mice that have not yet been identified in human studies. For example, we found strong linkage of two genetic loci on chromosome 5 (Fig. 4) containing Ncapg and Lcorl (chromosome 5: 46.03–46.48 Mb) and Bmp3 and Prkg2 (chromosome 5: 99.02–102.0 Mb). Human GWAS have found that these genes are strongly associated with human adult height^31^,^32^. Since body length in mice has been correlated with rotarod performance for certain mouse strains^18^, it is possible that allelic variation at these loci control body length in CC mice and contribute to rotarod performance. In addition, sight-impairment has been shown to affect rotarod performance^18^. We did not measure sight-impairment in our CC strains, however, a human GWAS of age-related macular degeneration identified a QTL at human chromosome 4q12 containing the following genes: REST, C4orf14, POLR2B and IGFBP7^33^. This locus corresponds to mouse chromosome 5 and overlaps with a genetic locus with significant association with rotarod performance (chromosome 5: 75.87–82.15 Mb; Fig. 4). Similarly, we found Barhl2, which is required for the development of multiple retinal cell types^34^, contained within a small (0.39 Mb) genetic locus associated with rotarod performance. Together, these data suggest that at least some of our genetic loci associated with rotarod performance could control body length and eyesight, however, further work is needed to confirm this. Additionally, our study focused on protein coding genes within these regions, and it is possible that other genetic regulatory sequences (e.g. lncRNA or miRNA) within our association loci could contribute to body weight and rotarod performance.

In our study, we observed a significant overlap in QTL associated with rotarod performance and body weight. QTL exclusively associated with body weight also showed overlap with human GWAS of neurodegenerative and neurodevelopmental disorders and vice versa. Obesity affects glucose and energy metabolism of brain cells, resulting in neuroinflammation in the brain and impaired neuronal function^35^. Indeed, weight loss has been suggested as a protective course for Alzheimer’s and Parkinson’s disease^36^. Moreover, it was recently shown that midlife adiposity can predict earlier onset of Alzheimer’s disease^37^. Taken together, our data provides further evidence for a relationship between obesity and neurodegenerative disease, and demonstrates the utility of the CC mice for further investigations of the genetic control of these and other traits and diseases.

## Materials and Methods

### Mice

Collaborative Cross mice were obtained from UNC Chapel Hill. Genotyping data was obtained from the UNC Systems Genetics Core website (http://csbio.unc.edu/CCstatus/index.py), which used Mouse Universal Genotyping Arrays containing 77.8 K markers (megaMUGA). Mice were acclimated at LBNL for eight weeks prior to the initiation of breeding. The study was carried out in strict accordance with the Guide for the Care and Use of Laboratory Animals of the National Institutes of Health. The animal use protocol was approved by the Animal Welfare and Research Committee of the Lawrence Berkeley National Laboratory (Protocol File Number 271004). All mice were weaned at 21 days and group housed whenever possible. Body weight and rotarod performance were measured at ten weeks of age. For details on the numbers and sex of mice used for each strain refer to Table S1.

### Rotarod performance

Mice were tested for their ability to maintain themselves upright on a rotating rod. Animals were placed on a spindle at 45-degree angle and subjected to a slow-speed “waiting” mode (4 rpm for 5–10 seconds) before acceleration. To avoid passive rotation of the mice on the rod we used a spindle with a diameter of 30 mm and a lane dimension of 50 mm. Acceleration was started after the “waiting” period and was set at 20 rpm/min for all mice. Trials where mice fell off in less than five seconds are likely due to operator error and were repeated and not included in the analysis. Animal falls were detected by a pressure sensitive lever, which automatically stops and records the speed at the time of the fall. All testing was conducted during the animal’s light cycle. All mice were tested consecutively an average of 3.3 times (range: 3–5), and repeat measurements of speed at the time of animal falling were averaged.

### Linkage analysis

At each SNP, phenotypic values (weight, rotarod performance) from individual CC mice were assigned to their respective alleles. We used logistic regression to regress phenotypic trait values on alleles at each SNP^38^,^39^. We computed p-values for the contribution of each allele to each phenotypic trait in the logistic model. We applied a false discovery rate (FDR) correction to the p-values for the estimated coefficients. SNPs with an FDR of p<1E-18 were called as significant, while those with FDR 1E-18<p<1E-8 were classified as suggestive^40^. QTL loci were defined as loci where at least 3 adjacent or closely clustered SNPs reached significance, and boundaries were defined by non-significant SNPs flanking significant loci. Suggestive loci were only identified for regions where either body weight or rotarod performance showed significant association. Putative candidate genes were defined as those genes (NCBI37/mm9 freeze) partially overlapping or contained within a QTL locus. The list of candidate genes was compared to human GWAS of neurological disorders and body weight-related phenotypes downloaded from www.ebi.ac.uk/gwas (see Table S4 for details).

## Additional Information

**How to cite this article**: Mao, J.-H. *et al.* Identification of genetic factors that modify motor performance and body weight using Collaborative Cross mice. *Sci. Rep.* **5**, 16247; doi: 10.1038/srep16247 (2015).

## Supplementary Material

Supplementary Information

Supplementary Table S1

Supplementary Table S2

Supplementary Table S3

Supplementary Table S4
