# Supplementary material for: Identification of genetic factors that modify motor performance and body weight using Collaborative Cross mice
Source: Sci Rep. 2015 Nov 9;5:16247. doi: 10.1038/srep16247 (PMC4637864; doi:10.1038/srep16247)
Supplement: Supplementary Information [file srep16247-s1.pdf]

Identification of genetic factors that modify motor performance and body weight using Collaborative Cross mice.

- Supplementary Information -

Jian-Hua Mao[1]*, Sasha A. Langley[1], Yurong Huang[1], Michael Hang[1], Kristofer E. Bouchard[1], Susan E. Celniker[1], James B. Brown[1], Janet K. Jansson[2], Gary H. Karpen[1,3] and Antoine M. Snijders[1]*

[1]Life Sciences Division, Lawrence Berkeley National Laboratory, Berkeley, CA, USA.

[2]Biological Sciences Division, Pacific Northwest National Laboratory Richland, WA, USA.

[3] Department of Molecular and Cell Biology, University of California, Berkeley, CA, USA.

*Correspondence to: Antoine M Snijders, E-mail: AMSnijders@lbl.gov, or Jian-Hua Mao, E-mail: JHMao@lbl.gov

Supplementary Materials

Figure S1. Correlation of rotarod performance and body weight between male and female mice.

The average rotarod performance for male and female mice of each strain (left; Pearson $r=0.94$, $p<0.0001$) and body weight for male and female mice of each strain (right; Pearson $r=0.86$, $p<0.0001$) are significantly correlated.

Figure S2. Human relevance of mouse genetic loci associated with rotarod performance and body weight.

Candidate genes within mouse genetic loci showed significant overlap with the genes associated with human body weight-related phenotypes and neurological disorders.

Table S1. Body weight and rotarod performance in male and female Collaborative Cross mice.

Table S2. Linkage analysis of body weight and rotarod performance.

Table S3. Candidate genes in genetic loci associated with body weight and rotarod performance.

Table S4. Human genome-wide association studies of neurological and body weight related disorders and traits.

Figure S1

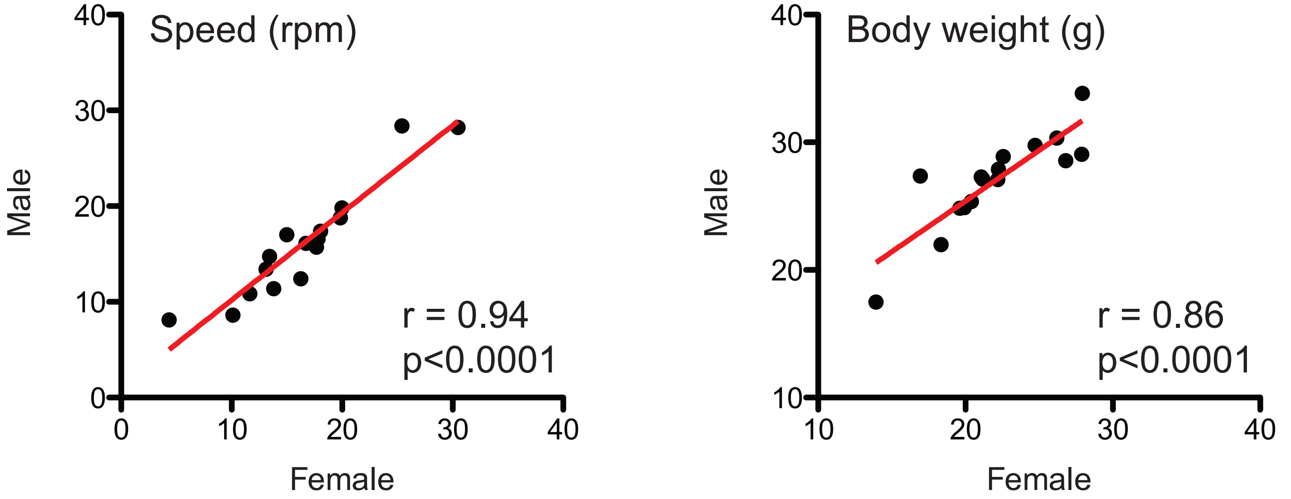
Speed (rpm)
r = 0.94
p<0.0001
Male
Female
0
10
20
30
40
Body weight (g)
r = 0.86
p<0.0001
Male
Female
10
20
30
40

Figure S2.

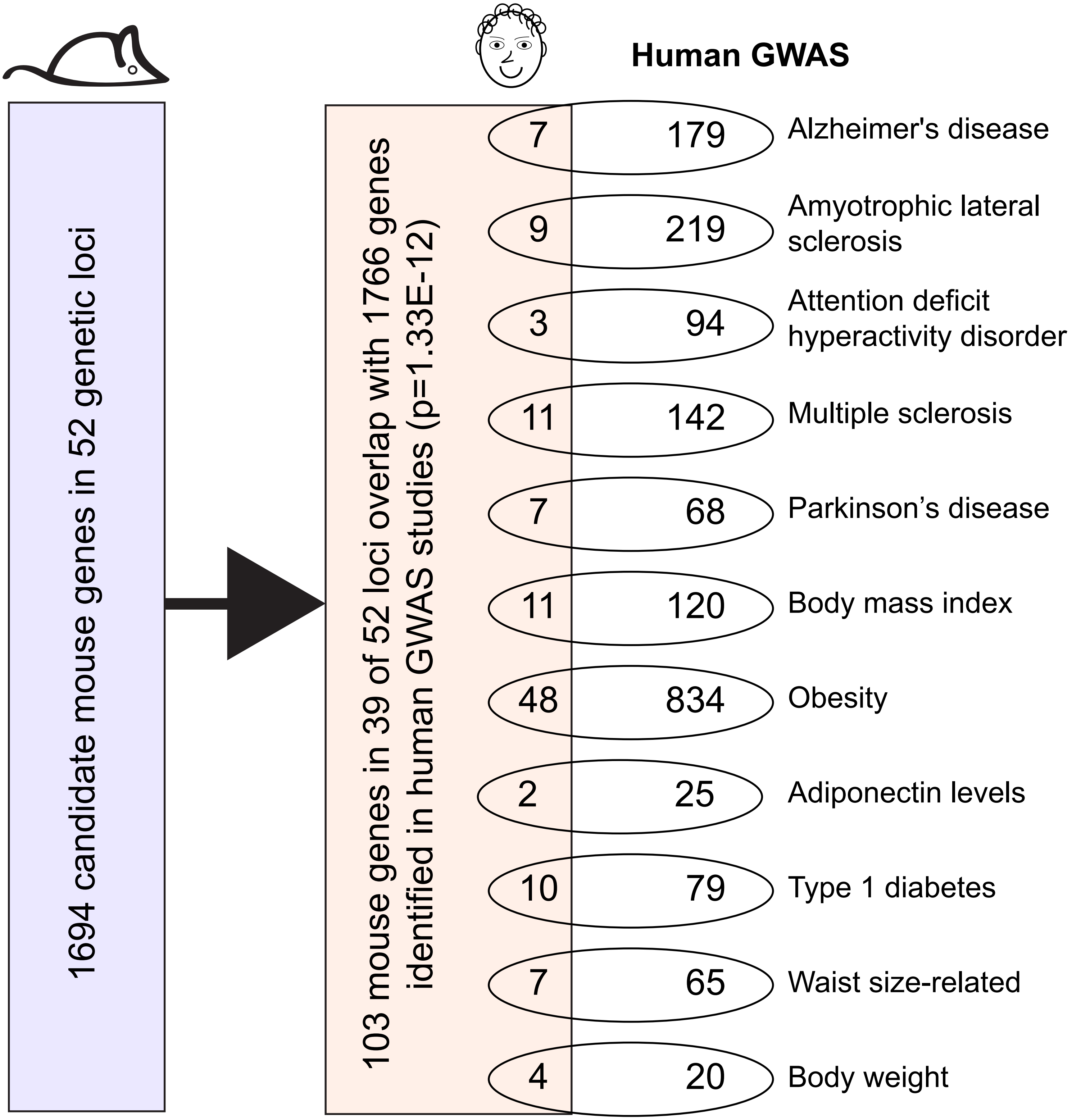
1694 candidate mouse genes in 52 genetic loci
103 mouse genes in 39 of 52 loci overlap with 1766 genes identified in human GWAS studies (p=1.33E-12)
Human GWAS
7
179
Alzheimer's disease
9
219
Amyotrophic lateral sclerosis
3
94
Attention deficit hyperactivity disorder
11
142
Multiple sclerosis
7
68
Parkinson's disease
11
120
Body mass index
48
834
Obesity
2
25
Adiponectin levels
10
79
Type 1 diabetes
7
65
Waist size-related
4
20
Body weight
